# Curating and Exhibiting for the Pandemic: Participatory Virtual Art Practices During the COVID-19 Outbreak in China

**DOI:** 10.1177/2056305120948232

**Published:** 2020-07-31

**Authors:** Xiaodan Feng

**Affiliations:** Vrije Universiteit Amsterdam, The Netherlands

**Keywords:** China, COVID-19, digital in-between space, online exhibitions, participatory art, state media discourse

## Abstract

Since China’s lockdown of major cities in response to COVID-19, different forms of online participatory initiatives led by self-organizing groups of volunteers have greatly contributed to information circulation, patient admission support, and other aspects of coping with the pandemic. Although often overlooked by those studying online cultural production during the pandemic, a massive spontaneous and participatory creative outpouring of individual and collaborative artworks related to “fight the pandemic” are being published through platforms including Kuaishou, TikTok, and WeChat public accounts. This article argues that while these participatory online exhibitions published through WeChat opened up a temporary space of expression that both offset the lack of information and enabled alternative ways of understanding of and expression about the crisis, they were not only subject to pervasive state surveillance, but also co-optation by state media.

Since the COVID-19 outbreak in China, the government has been producing a ceaseless flow of messages initiated by Chairman Xi Jinping, which depicts the pandemic as *renmin zhanzhen*人民战争 “people’s war,” *zongtizhan*总体战 “total war,” and *zujizhan*阻击战 “blockade.” This wartime narrative is part of an overarching communication effort by Chinese state media committed to the production of constant, conformity, and perfunctory positive imagery. These efforts aim to achieve and justify the party-state’s exceptional command and control in the mobilization of all sorts of resources during the pandemic and induce nationalistic sentiments among the public by implying the disease as the invasive “other” in opposition to the party-state.

At the same time, there has been an outpouring of various kinds of participatory virtual art under the theme of “fight the pandemic” published on platforms including Kuaishou, TikTok, and WeChat. This participatory artistic expression offers an alternative to the top-down government narratives, giving people access to the means of constructing and sharing narratives around their own experiences during the pandemic, enabling the public to understand the crisis differently than the perspective propagated by the state media. The power and impact of these artistic practices, however, was undercut in part by the government usurping their power in the service of party-state’s ideological and aesthetic repertoire.

## Overview of the State Media Messaging

The two dominating characteristics of the Chinese state media messaging during the pandemic are the adoption of the military language and the construction of a one-sided positive image of the government’s efforts and accomplishments. The military language of pandemic, initiated by Chairman Xi Jinping, frames the disease as the enemy of the entire national population, which should be fought against by exhausting all efforts and any form of sacrifice. For example, upon conducting simple search queries of the military metaphor: *yiqing fangkong zujizhan*疫情防控阻击战 “anti-pandemic blockage” through the built-in search engines of the three major state owned-media sites People’s Daily, Xinhua News Agency, and China Central Television, the results returned are 10,659, 10,000,^1^ and 19,555 results, respectively (for articles containing this term either in the title or content). Similar metaphorical terms like *renmin zhanzhen*人民战争 “people’s war” and *zongtizhan*总体战 “total war” also return a significant number of results. In addition, variations on these phrases in the forms of slogans and song lyrics have been displayed on street banners, digital screens, and billboards, making war narrative ubiquitous.

A one-sided positive image is constructed by the government mainly through hero-making by excessively glorifying and eulogizing frontline workers to propagate the notion of great leadership by the state. Media events that glamorize frontline stories such as *zhanyi yingxiong gushihui*战疫英雄故事会 “Story-Sharing Session of the Heroes of the Pandemic” are being organized by local-level party units, with the aim not only to justify the party-state’s exceptional control and command of resources during the pandemic but also to shift focus away from its early concealment of information on COVID-19.

## Overview of the Online Participatory Art Practices

**Figure 1.**
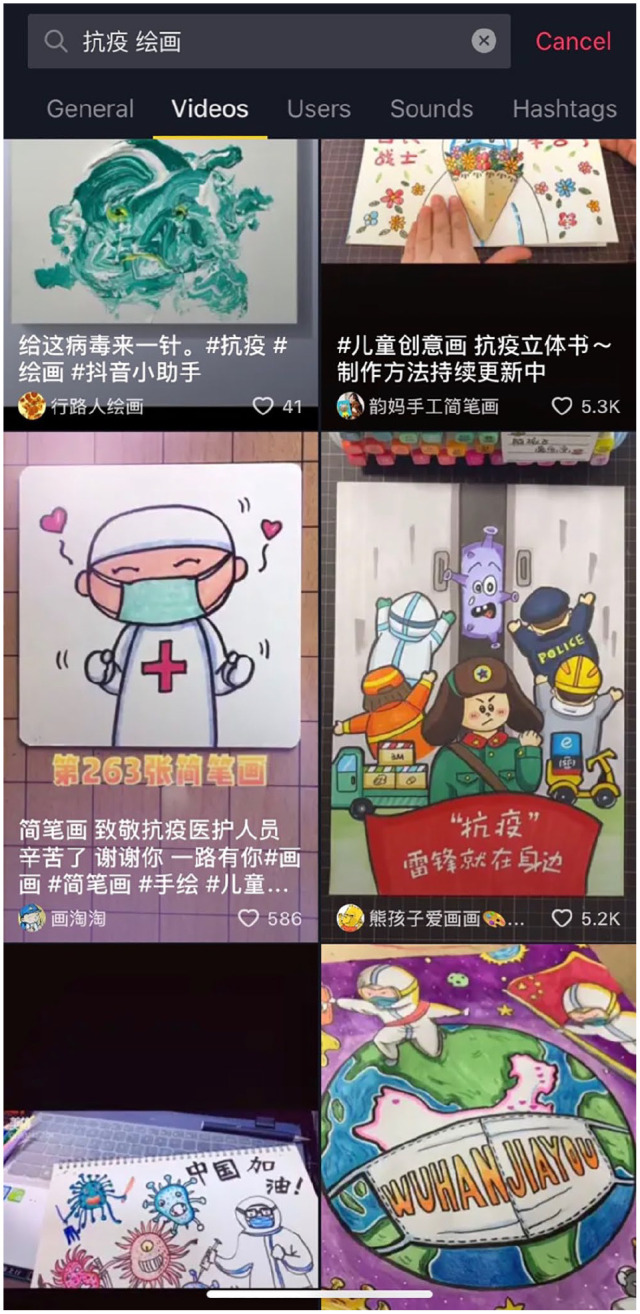
Screenshot of TikTok’s interface when searching “kangyi抗疫” (anti-pandemic) and “huihua绘画” (painting) (April, 2020).

A contrasting scene, however, is produced through online participatory art practices, the majority of which are published through Kuaishou, TikTok, and WeChat public accounts. For Kuaishou and TikTok, the artworks are posted individually in the form of short videos (see Figure 1). The top searches on TikTok^2^ under the “anti-pandemic” tags are for paintings, posters, clippings, and music postings. These postings aim to convey morale-boosting messages through instructional demonstration of composition processes such as drawing cartoon subjects like doctors or virus molecules and delivering them with compelling subtitles and background music. Seemingly unremarkable at first glance, these postings demonstrate sincerity and genuineness in expressing concern, care, and support toward the COVID-19 frontline workers. They represent the concrete lived experience, the “everyday life” which offer resistance to the discourse being championed by the ruling institutions (De Certeau, 1984; Lefebvre, 2008).

**Figure 2.**
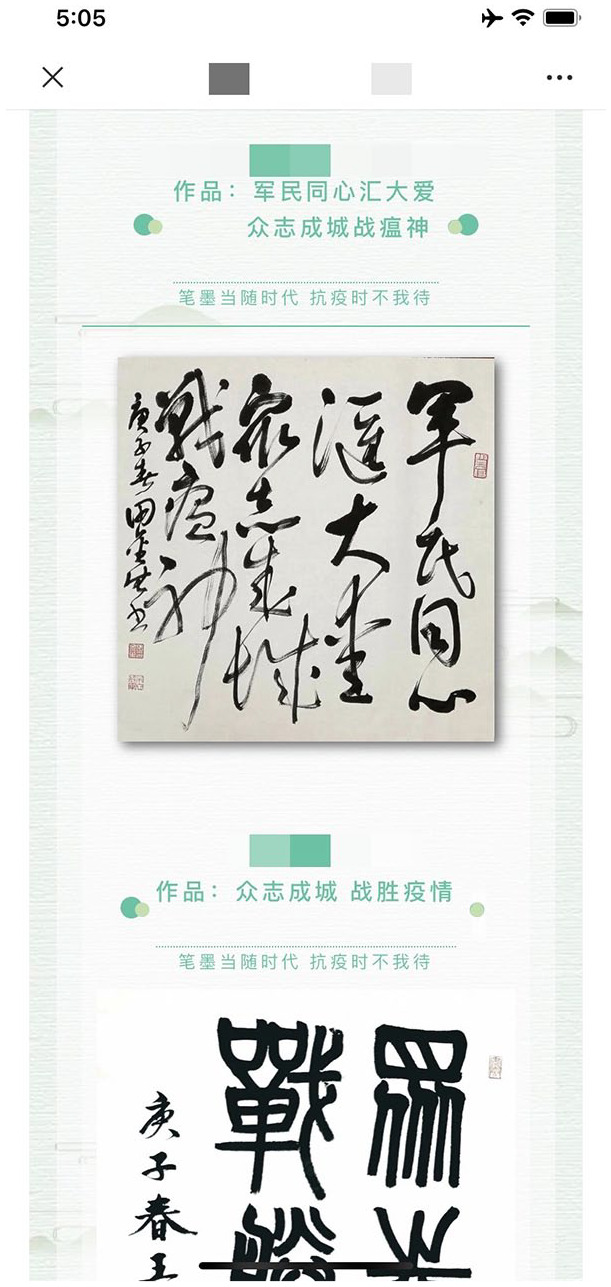
Screenshot of WeChat’s interface of a random online exhibition (May, 2020).

The online exhibitions published through WeChat are different in format and are larger in scale. The enormity of the body of work produced, and the frequency with which they are shared, make the art posted on WeChat a more powerful counter to state messaging. When these online exhibitions first appeared in January, most of them were organized and posted on WeChat public accounts by so-called grassroots organizations, such as interest-based societies, clubs, and communities (most often relating to poetry, calligraphy, and other crafts). By February, there were frequent posts by more official organizations such as schools and private art institutes or academies, nongovernmental organizations (NGOs), as well as artists associations, public culture centers, and social service organizations of the district, city, or province. Each exhibition contains approximately 10–20 pieces of artwork solicited. By searching “*kangyi*抗疫 (anti-pandemic)” and “*zhanlan*展览 (exhibition)” through WeChat, tens of thousands of such postings appear in the public domain (see Figure 2 for example). The contents of these exhibitions are varied, including different styles of paintings, calligraphy works of poems and personal diaries, Chinese paper cutting, woodcutting, and many more. Under the theme of *kangyi*, some artworks embody rich elements of localism and metaphors, extending the discursive power of commonly exhibited objects by referencing local communities; some of the artworks compensate for the situation of media immobility during the lockdown with their portrayal of frontline scenes; others take the form of feminist or LGBT advocacy, attempting to interweave the activist discourse (which normally lacks coverage) with the overarching pandemic narrative under political homogeneity.

## Participatory Online Exhibitions as a Digital Space In-Between

**Figure 3.**
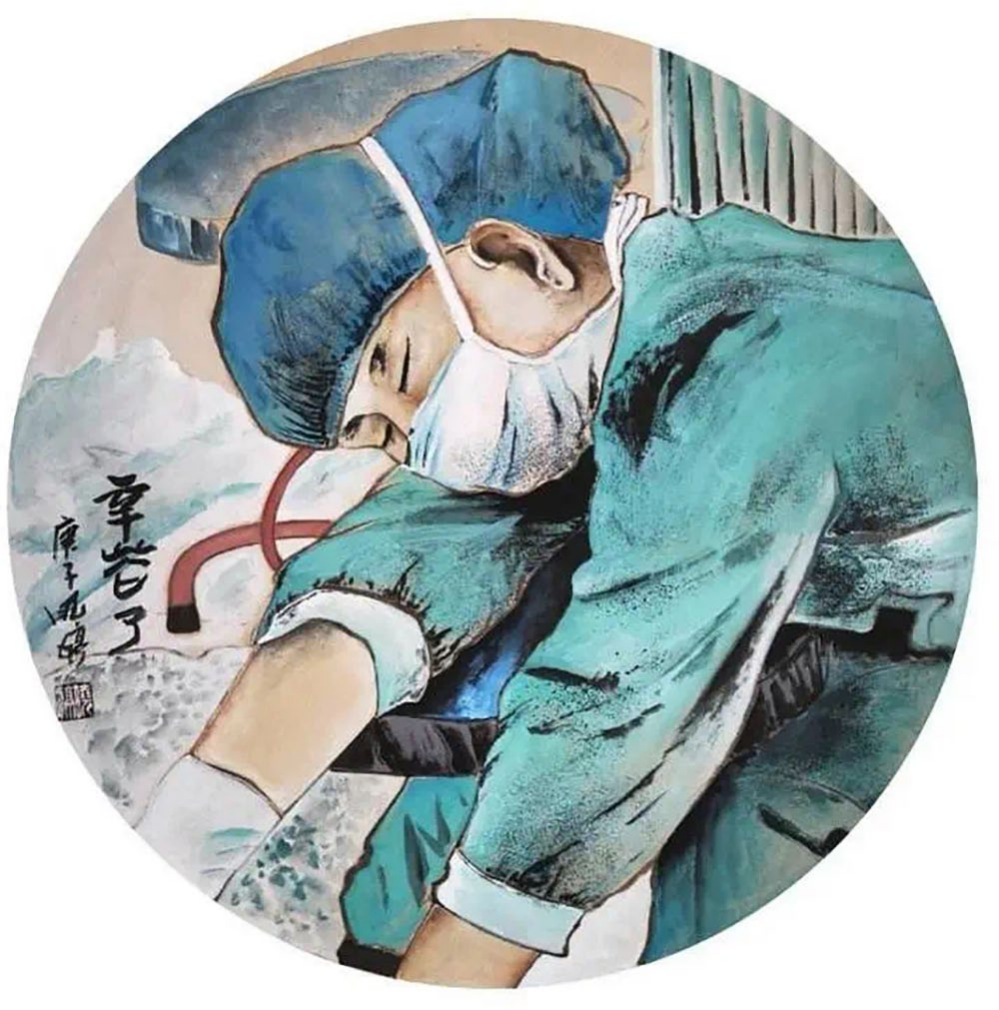
A female medical worker taking a nap. From “辛苦了” (Thank You for Your Hard Work) by X. Dang (February, 2020).

**Figure 4.**
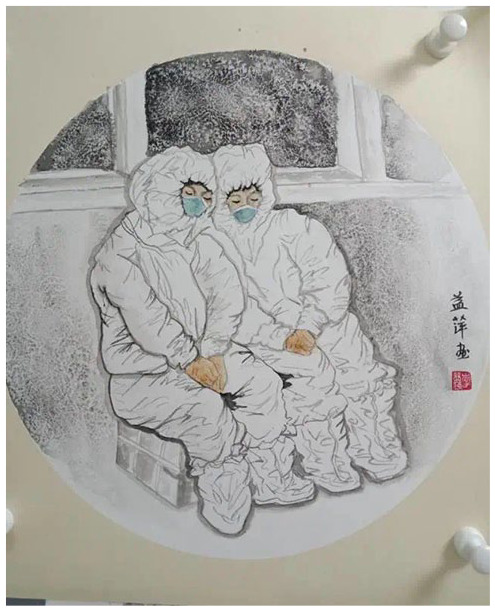
Female medical workers sleeping in the hallway. From “枕戈待旦” (Working Overnight) by Y. Li (February, 2020).

**Figure 5.**
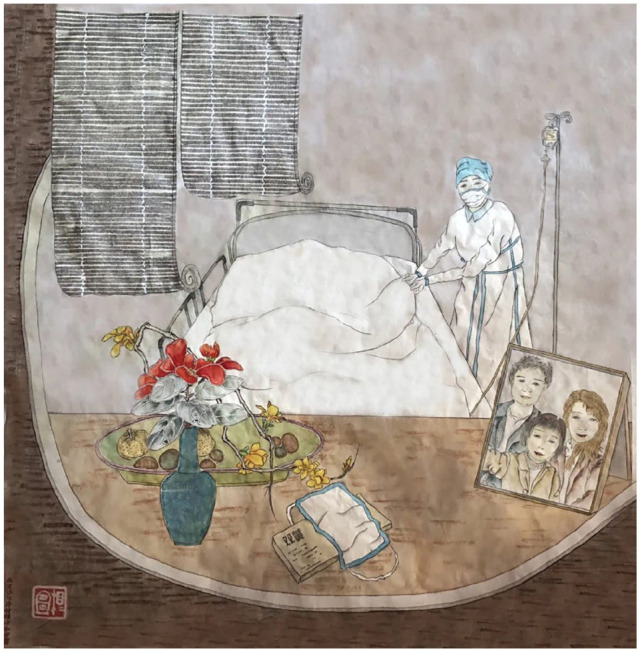
A female medical worker taking care of the patient, with a family photo of her placed on the table. From “医者” (The Medical Worker) by X. Dang (March, 2020).

Against the backdrop of government messaging, people are able to share their experience and emotions genuinely through these online exhibitions, and in so doing they produce alternative media that is more informative, conscientious, and consoling than the state media (Moreno-Almeida, 2020). For example, a video posted by the Gansu provincial party-run *Gansu Daily* showing female medical workers having their heads shaved before going to support Hubei Province^3^ ignored the fact that some of them are crying and may have been forced, and promoted it instead as evidence of heroic. Such toxic (or cruel) positivity aroused public anger for, as one editorial put it, “humiliating women and using them as tools for propaganda” (Chen, 2020). At the same time, many online exhibitions were explicitly devoted to the praise and recognition of female medical workers: they gathered and displayed works which portrayed actual working conditions of medical staff, as well as the expression of the artists’ sincere well-wishes (see Figures 3 to 5). For example, the image of a female medical worker depicted in Figure 3 was taken from an online exhibition which followed the theme of praising females’ efforts in the pandemic fight (*jinguo kangyi*巾帼抗疫^4^), organized by the public account “The Voice of Women of Weinan City” (*Weinan nüsheng*渭南女声, 2020). The image portrays a female medical worker taking a nap, with the line “thank you for your hard work” (“辛苦了”) on the left side of the image. Another two examples, Figures 4 and 5, were taken from a similar exhibition organized by the public account of “Heroines of Guilin City” (*jinguo Guilin*巾帼桂林), and similarly portray in detail the normal scenes of working female medical workers. Besides the paintings, there are many calligraphy works of poems and diaries which express appreciation and praise to the female medical workers for their efforts. Both exhibition postings show respect and appreciation with a sincerity that quietly contrasts the state depictions, exhibiting what Burgess (2006, p. 207) calls “vernacular creativity [of] everyday cultural production.”

**Figure 6.**
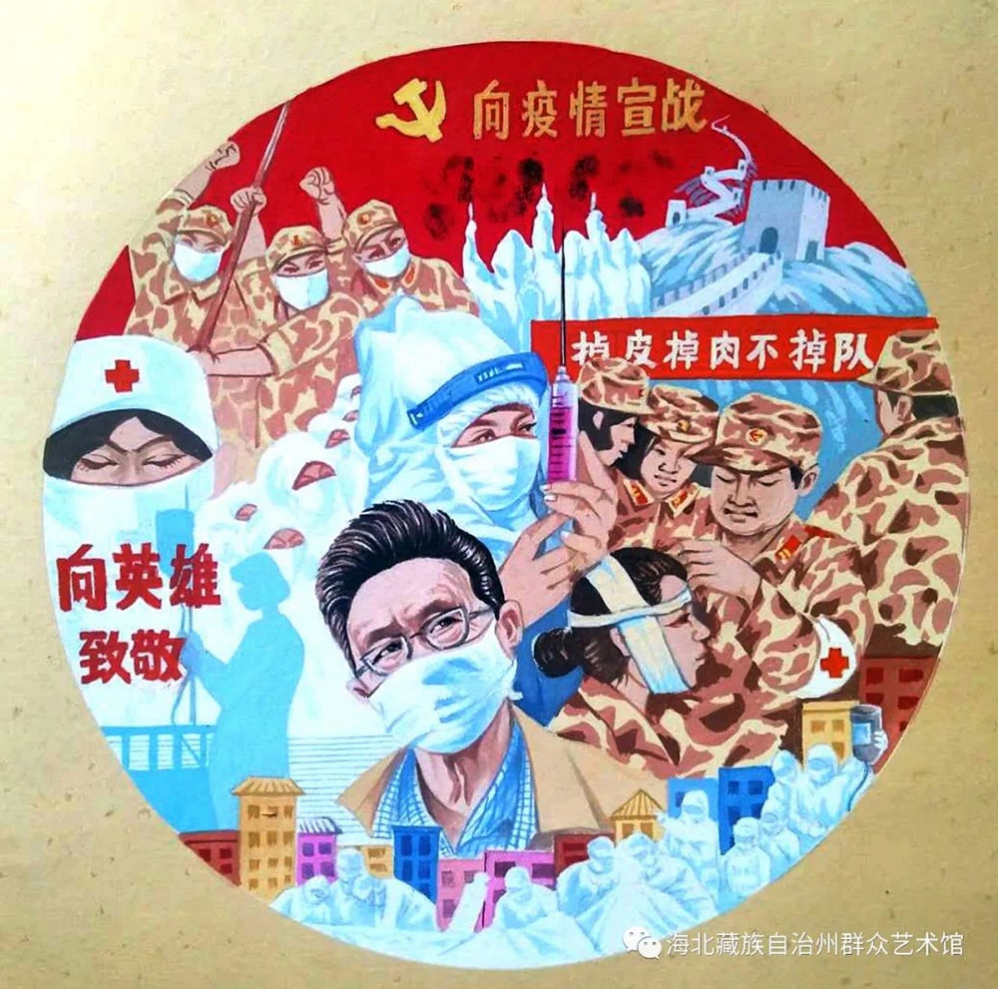
Doctors, liberation army, “declare war against the pandemic.” From “向英雄致敬” (A Tribute to Heroes) by S. Chai (April, 2020).

**Figure 7.**
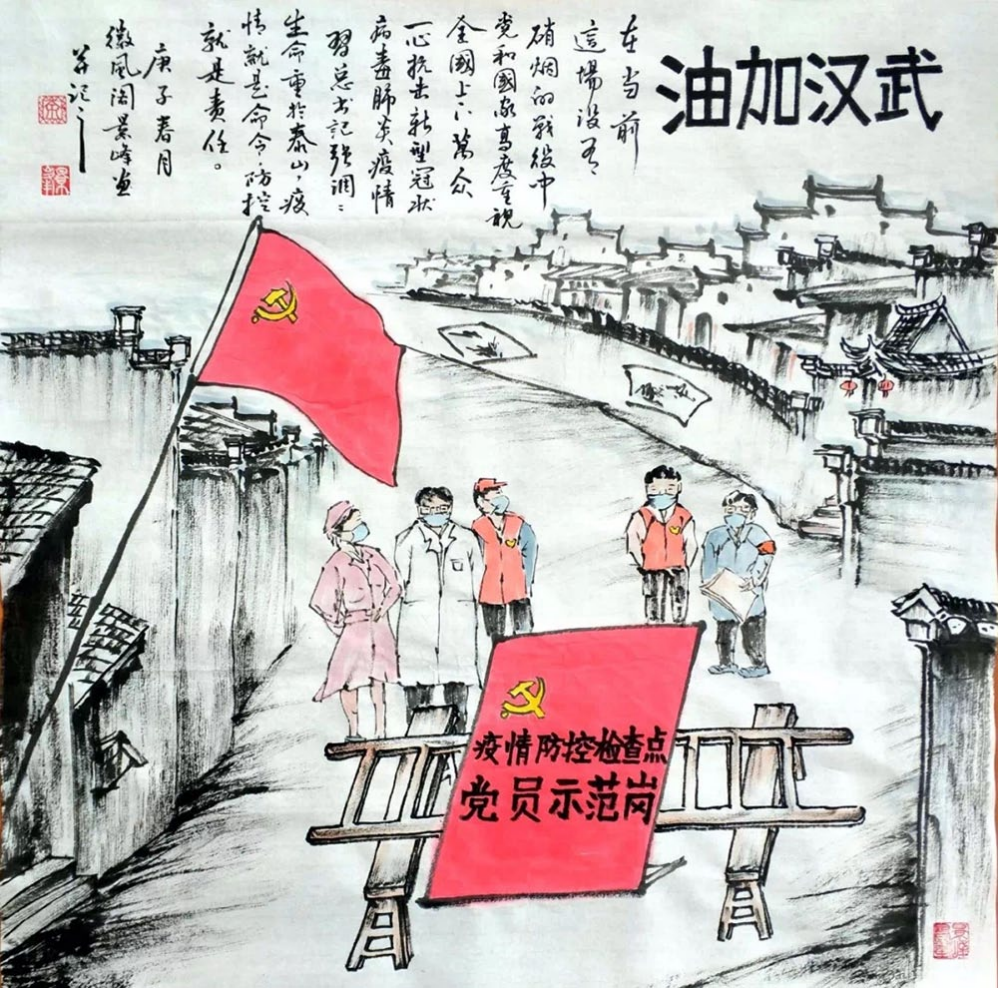
Huizhou landscape, doctors, and local cadres of the Communist Party. From “武汉加油” (Wuhan, Jiayou) by J. Yu (February, 2020).

**Figure 8.**
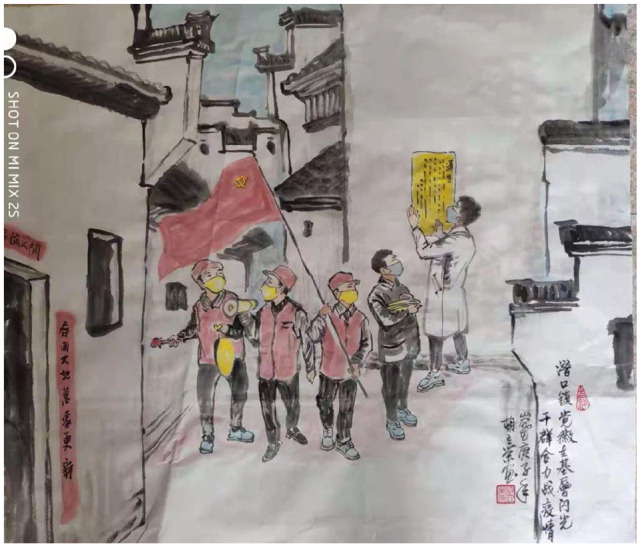
As above. From “潜口镇党徽在基层闪光” (The Illuminating Efforts of Qiankou Town Grassroots Party Officials) by L. Hu (February, 2020).

**Figure 9.**
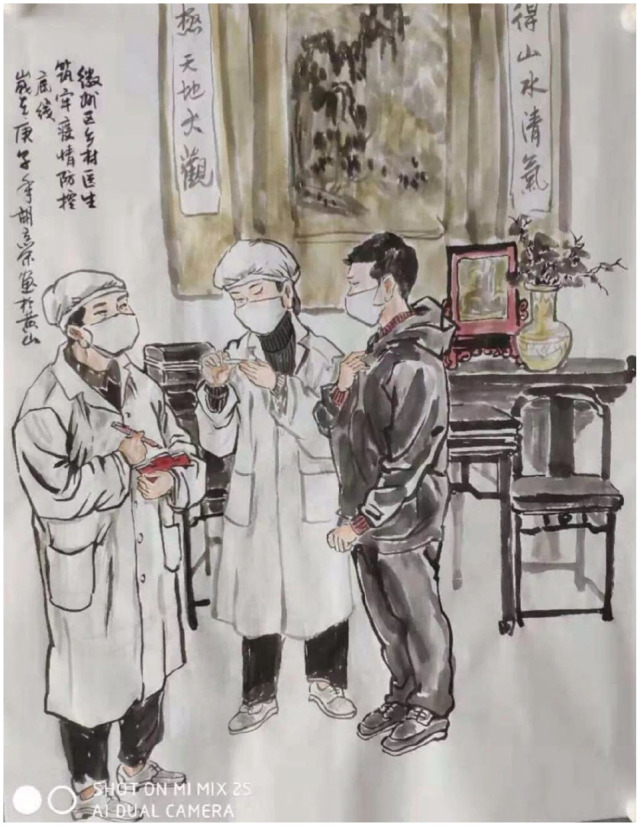
Doctors visiting households. From “徽州区乡村医生” (Village Doctors from the Huizhou District) by L. Hu (February, 2020).

At the same time, with Internet censorship becoming increasingly stringent, the aforementioned Chinese online participation is also subject to pervasive intrusion of state narratives and intervention. The Chinese term *shoubian*收编 specifically denotes something that was originally not a part of but eventually being co-opted by the Chinese Communist Party (CCP) (possibly because of being censored) and serve their ideological purpose from then on. These participatory art practices too, are gradually being “infected,” being *shoubian* by the government in suppressing their power as a counter-hegemonic force. By the end of February, the state’s constant effort to maintain the “wartime narrative” and to push CCP ideologies are seen in more and more of the paintings. The subjects drawn begin to appear as heroes, sometimes appear alongside propagandistic or even Maoist expressions. Government operatives (from the propaganda department) call up their forces from higher to lower levels of party units, to communicate those ideological expressions through contributions to these online art spaces. For example, there was an entire section of *China Art News*^5^ on 24 February 2020 devoted to the discussion of artworks’ accountability in communicating patriotism during the pandemic. On the contrary, the rampant spread of the virus also arouses a shared sympathy toward the government’s radical approach to containing the COVID-19. Echoing de Kloet et al. (2020), this shared sympathy and understanding among the Chinese public has given rise to the “biopolitical nationalism,” in which “the biopolitical efforts of nation-states are being compared, applauded and supported.” Some people’s self-censoring, loyalty-showing, or simply automatic internalization of such imaginary and language also partly contribute to the status quo. Figure 6 is taken from the online exhibition published by the public account of Haibei Tibetan Autonomous Prefecture Mass Art Center (*Haibei zangzuzizhizhou qunzhong yishuguan*海北藏族自治州群众艺术馆, 2020) which explicitly combines current issues with elements typical of the Communist Revolution, namely the image of the People’s Liberation Army and the familiar slogan which translates to “get wounded and do not lag behind.” Similarly, the images in Figures 7 to 9, published by the account of “Huizhou Micro-tourism” (*Huizhou weilüyou*徽州微旅游, 2020), depict traditional Huizhou architecture, landscape, and lifestyle, some of which include the presence of the Communist Party flag and local party members on patrol during the pandemic. These images show that the governmental narratives and ideologies dominate. In the face of such interference, the participatory efforts seem to strengthen rather than undermine government propaganda (Morozov, 2011, p. 14). This practice of appropriating grassroots online practices and cultural forms is common among authoritarian governments.

So, on one hand, these creative online exhibitions convey the messages, information, and narratives that are so often ignored by state media, constituting a moderating apparatus—potentially “a new form of oppositional intelligence” (Kester, 2011, p. 44). On the other hand, these participatory and alternative practices do not fall completely out of the state governance, demonstrated by their reuse of official narratives and language. These participatory exhibitions and artworks collectively produced a peculiar creative digital in-between space, in the sense that they appear as neither the “radical protest” nor the “diffused contention,” the forms of counter-hegemony practices through Chinese media elaborated by Guobin Yang (2013). Instead, they opened up the temporal space of expression that overtime was appropriated by the state, but that nevertheless carved out a space for alterative imaginations of the crisis. This “alternative” does not equate to a bold, direct critique in opposition to the state; it is instead a means through which quotidian and seemingly mundane contributions pervade public compassion more strongly than a contrived narrative can.

## Notes

1. It seems that 10,000 is the upper limit for the Xinhua News Agency site to return its searching results, as I have also searched randomly several other very commonly used terms, and the number of results consistently shows 10,000.

2. TikTok as referred to throughout this essay is the Chinese version.

3. Hubei’s provincial capital Wuhan is believed by many experts to be where the pandemic outbreak began. Hubei Province was the most severely affected area in China during the outbreak.

4. 巾帼(*jinguo*) originally denotes the head scarf of ancient Chinese women; its meaning now is closer to “heroines,” a term of high respect and recognition for working females and their contribution.

5. China Art News is run by the China Federation of Literary and Art Circles (CFLAC) and is considered as the most important and authoritative newspaper in the area of art and culture in China.
